# Phylogenomics and Evolutionary Dynamics of the Family *Actinomycetaceae*

**DOI:** 10.1093/gbe/evu211

**Published:** 2014-09-22

**Authors:** Kelei Zhao, Wujiao Li, Chunlan Kang, Lianming Du, Ting Huang, Xiuyue Zhang, Min Wu, Bisong Yue

**Affiliations:** ^1^Key Laboratory of Bio-resources and Eco-environment (Ministry of Education), College of Life Sciences, Sichuan University, Chengdu, China; ^2^Department of Basic Sciences, School of Medicine and Health Sciences, University of North Dakota; ^3^Sichuan Key Laboratory of Conservation Biology on Endangered Wildlife, College of Life Sciences, Sichuan University, Chengdu, China

**Keywords:** *Actinomycetaceae*, *Trueperella pyogenes*, comparative genomics, phylogeny, gene gain/loss

## Abstract

The family *Actinomycetaceae* comprises several important pathogens that impose serious threat to human health and cause substantial infections of economically important animals. However, the phylogeny and evolutionary dynamic of this family are poorly characterized. Here, we provide detailed description of the genome characteristics of *Trueperella pyogenes*, a prevalent opportunistic bacterium that belongs to the family *Actinomycetaceae*, and the results of comparative genomics analyses suggested that *T. pyogenes* was a more versatile pathogen than *Arcanobacterium haemolyticum* in adapting various environments. We then performed phylogenetic analyses at the genomic level and showed that, on the whole, the established members of the family *Actinomycetaceae* were clearly separated with high bootstrap values but confused with the dominant genus *Actinomyces*, because the species of genus *Actinomyces* were divided into three main groups with different G+C content. Although *T. pyogenes* and *A. haemolyticum* were found to share the same branch as previously determined, our results of single nucleotide polymorphism tree and genome clustering as well as predicted intercellular metabolic analyses provide evidence that they are phylogenetic neighbors. Finally, we found that the gene gain/loss events occurring in each species may play an important role during the evolution of *Actinomycetaceae* from free-living to a specific lifestyle.

## Introduction

The family *Actinomycetaceae* was originally identified in 1918 (Buchanan 1918) and was the only member of the order *Actinomycetales* following the update of 16S rRNA signature nucleotide patterns (Zhi et al. 2009). The membership of this family had been changed for several times along with the improvement of taxonomic techniques, such as phenotypic characteristics, chemotaxonomic, numerical phenetic, and molecular genetic procedures (Slack 1974; Schaal et al. 2006). As currently defined, the family *Actinomycetaceae* comprises six valid genera including *Actinomyces*, *Actinobaculum*, *Varibaculum*, *Mobiluncus*, *Arcanobacterium*, and *Trueperella* (Collins, Jones, Kroppenstedt, et al. 1982; Spiegel and Roberts 1984; Lawson et al. 1997; Stackebrandt et al. 1997; Hall et al. 2003; Schaal et al. 2006; Yassin et al. 2011). Most of the members have been exclusively found as commensals or pathogens of humans and warm-blooded animals.

Although there were increasing studies concerning the accession and classification of new species in *Actinomycetaceae* during the past century, the current taxonomy might not satisfy the interests of related researchers due to the limited knowledge of genomic information. The established phylogenetic patterns of *Actinomycetaceae* were primarily dependent on the sequence analyses of 16S rRNA; however, single-gene-based phylogenetic analyses may provide limited phylogenetic information and reliable additional differential characteristics are hardly available (Schaal et al. 2006; Funke et al 2010; Yassin et al. 2011). For example, the species of the genus *Actinomyces* can form different phylogenetic clusters depending on the cutoff branching points, as the bootstrap values of branching are fairly low (Schaal et al. 2006). Additionally, the generic name of *Trueperella pyogenes* has been changed at least four times (Cummins and Harris 1956; Barksdale et al. 1957; Collins and Jones 1982; Collins, Jones, Schofield, et al. 1982; Ramos et al. 1997; Yassin et al. 2011). Here, we provided the genome sequence of *T. pyogenes*, in combined with that of related lineages, to provide the genomic level insight into the classification and genome divergence during the evolution of the family *Actinomycetaceae*.

### Genome Sequencing and Analyses of *T. pyogenes* TP8

**Fig. 1.—:**
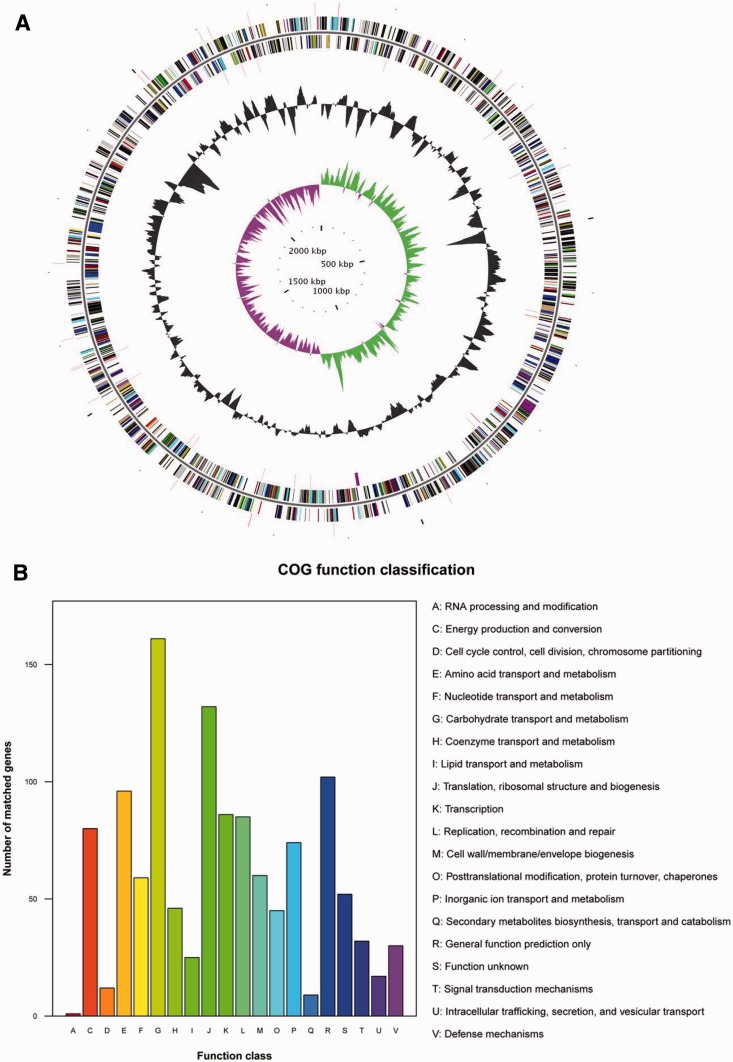
General features of *T. pyogenes* TP8. (*A*) Graphical circular map of the genome showing (from outside to centre): Genes on forward strand (color by COG categories), genes on reverse strand (color by COG categories), RNA genes, GC content, GC skew. (*B*) COGs function classification of *T. pyogenes* TP8.

**Fig. 2.—:**
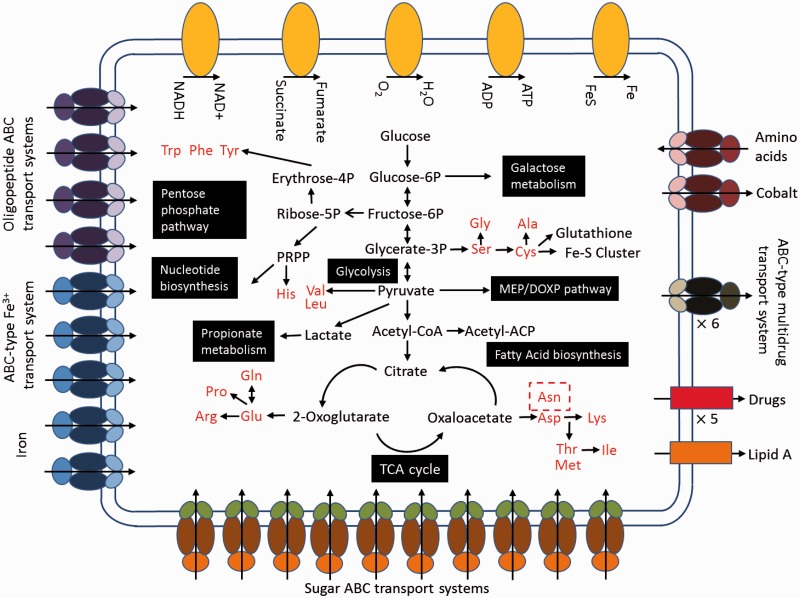
An overview prediction of the *T. pyogenes* TP8 metabolism and transport. The main elements of metabolic pathways in the *T. pyogenes* TP8 genome are shown in black. Amino acids are in red.

The *T. pyogenes* TP8 strain was isolated from the abscess of forest musk deer (Zhao et al. 2011) and was subjected to whole-genome sequencing. The genome was sequenced to greater than 15× coverage using the 454 sequencing platform, and then the high-quality sequence data were assembled into 14 contigs by Newbler2.6. A total size of 2.27-Mb genome sequence was subsequently acquired after the gaps were complemented by polymerase chain reaction (PCR) amplification (fig. 1*A* and supplementary table S1, Supplementary Material online). The coding DNA sequences contain 2,105 open reading frames (ORFs) with a total length of 2,045,904 bp covering about 90.03% of the whole genome after genome annotation. A Type 1 CRISPR (clustered regularly interspaced short palindromic repeats)/Cas (CRISPR-associated) system containing a long CRISPR array (30 repeats) was identified (supplementary fig. S1, Supplementary Material online). A total of 1,105 genes were affiliated with known Clusters of Orthologous Groups (COGs), of which 947 represented specific functional categories (excluding 106 “general function prediction only” and 52 “function unknown”) (fig. 1*B*). The integrated intracellular metabolic pathway of *T. pyogenes* TP8 was predicted based on the database of kyoto encyclopedia of genes and genomes (KEGG) (fig. 2 and supplementary fig. S2, Supplementary Material online). Critically, our result here was also confirmed by parallel analyses using the two recent reports of *T. pyogenes* genome sequences (Goldstone et al. 2014; Machado and Bicalho 2014). Additionally, *T. pyogenes* has one carbon pool by folate, which can be synthesized through nucleotide biosynthesis (supplementary fig. S3, Supplementary Material online).

### Comparative Genomic Analyses between *Arcanobacterium haemolyticum* and *T. pyogenes*

**Fig. 3.—:**
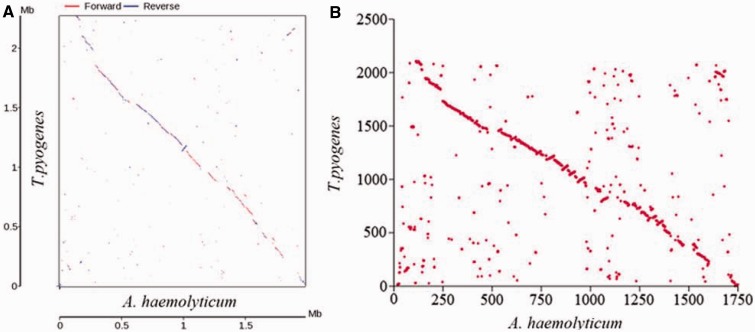
Comparative genomic analyses of *T. pyogenes* TP8 and *A. haemolyticum* DSM 20595. (*A*) Synteny analyses of *T. pyogenes* TP8 and *A. haemolyticum* DSM 20595 based on the nucleotide sequence. (*B*) Relative ORF positions of orthologs in *T. pyogenes* TP8 versus *A. haemolyticum* DSM 20595.

The classification between *Arcanobacterium haemolyticum* and *T. pyogenes* had been discussed for several decades. Previous studies used to suggest not only that both organisms were related to the *streptococci*, but also that *A. haemolyticum* should be a mutant form of *T. pyogenes* (Cummins and Harris 1956; Barksdale et al. 1957). In this study, our comparative genomics analysis between *A. haemolyticum* (Yasawong et al. 2010) and *T. pyogenes* showed that they have large areas of synteny in nucleotide sequence and relative ORF positions of orthologs (fig. 3). However, functional difference analyses suggested that *T. pyogenes* contains more ORFs (>300) than *A. haemolyticum,* covering almost all aspects of bacterial performance as confirmed by the gene ontology term analysis (supplementary fig. S4, Supplementary Material online). The result of KEGG pathway analysis showed that *T. pyogenes* has more advantages in metabolisms especially some amino acids and in causing human diseases (supplementary fig. S5, Supplementary Material online). Finally, the comparison of predicted global metabolic pathways of *A. haemolyticum* and *T. pyogenes* suggested that *T. pyogenes* has a more comprehensive intracellular metabolism system than that of *A. haemolyticum* such as lipid and amino acid metabolism (supplementary fig. S6, Supplementary Material online). These differences may be the genetic basis for why *T. pyogenes* can invade extensive host types and provide a plausible evidence for the distinction of *A. haemolyticum* and *T. pyogenes*.

### Phylogenomic Analyses of *Actinomycetaceae*

**Fig. 4.—:**
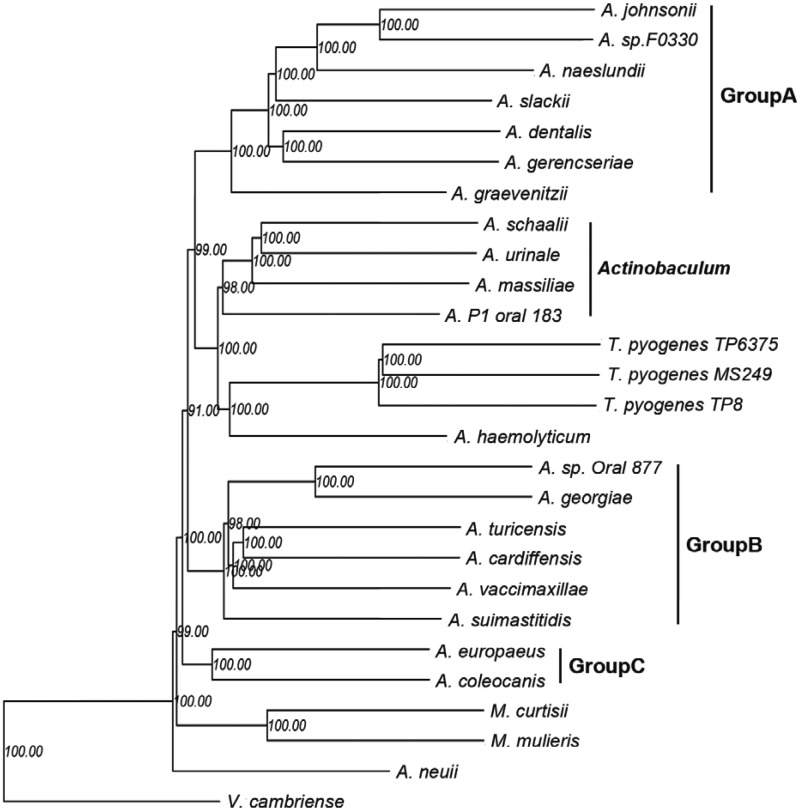
Common gene contents tree of the main species in the family *Actinomycetaceae*. The phylogenetic tree was constructed based on single-copy genes that simultaneously present in all species using ML estimation with a bootstrap value of 1,000 replications. All the 188 trees were merged by MP-EST in the website http://bioinformatics.publichealth.uga.edu/SpeciesTreeAnalysis/mpest/mpest.php (last accessed September 27, 2014) (Liu et al. 2010). The inferred numbers of genes presented in the main node (red dots) and each species are shown. Orange crooked arrow: gene gains. Green crooked arrow: gene losses.

The participation of *T. pyogenes* genomic sequence allows us to systematically analyze the evolution of *Actinomycetaceae* at the genomic level. After alignment of whole COGs, a total of 186 common genes with single copy in all taxa were obtained (supplementary data sheet S1, Supplementary Material online). The phylogenetic tree was then constructed using maximum-likelihood (ML) estimation based on the nucleotide sequences of the 186 ancestral genes (fig. 4). On the whole, the genera *Varibaculum*, *Actinobaculum*, and *Mobiluncus* could be readily distinguished from their closest phylogenetic relatives but may be indistinguishable from the dominant genus *Actinomyces*. Notably, the species of genus *Actinomyces* were divided into three main groups with high bootstrap values and different G+C content (supplementary table S2, Supplementary Material online). Therefore, our results here were convincing compared with the previous discussion on the classification of *Actinomyces* based on 16S rRNA analysis (Schaal et al. 2006). However, as previously determined, *A. haemolyticum* and *T. pyogenes* also shared the same branch (Schaal et al. 2006; Funke et al. 2010; Yassin et al. 2011). We then utilized a total of 267,240 identified single nucleotide polymorphisms (SNPs) to construct another phylogenetic tree and showed similar lineage classification with the COG tree (supplementary fig. S7, Supplementary Material online). Surprisingly, *A. haemolyticum* was apart from *T. pyogenes* although the bootstrap value was relatively low. We then searched for more information by performing whole COGs-based genome clustering (supplementary fig. S8 and data sheet S2, Supplementary Material online), the similarity coefficient of COG patterns between *T. pyogenes* and *A. haemolyticum* was lower than that between *T. pyogenes* and *A. coleocanis* and *A. europaeus*. Overall, our current results in conjunction with the previous analysis 16S rRNA and chemotaxonomic characteristics as determined by Yassin et al. (2011), indicated that *T. pyogenes* and *A. haemolyticum* should be classified into two different but highly phylogenetic related lineages.

### Functional Enrichment and Gene Gain/Loss Analyses

**Fig. 5.—:**
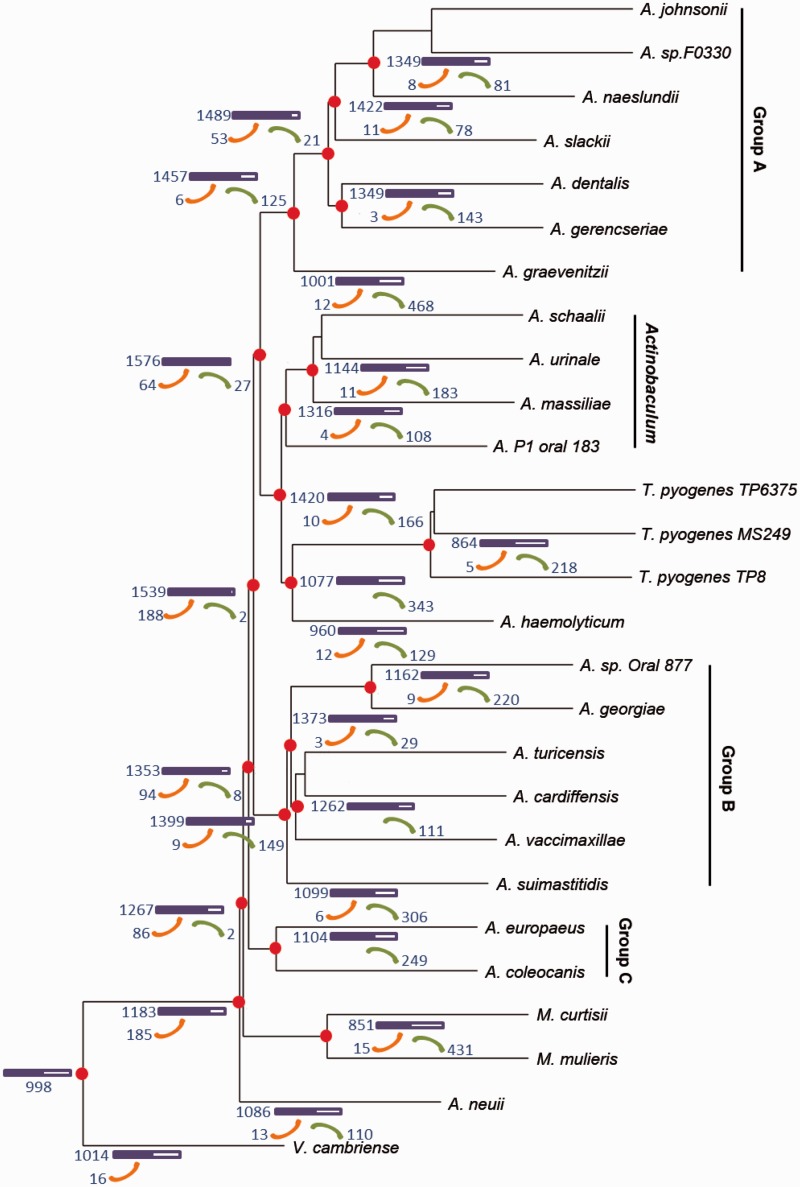
Gene gain and loss events in the evolution of the family *Actinomycetaceae*. The tree from figure 3 was used as a guide for the reconstruction. Purple frame, gene content; Orange arrow, gene gain; Green arrow, gene loss.

**Fig. 6.—:**
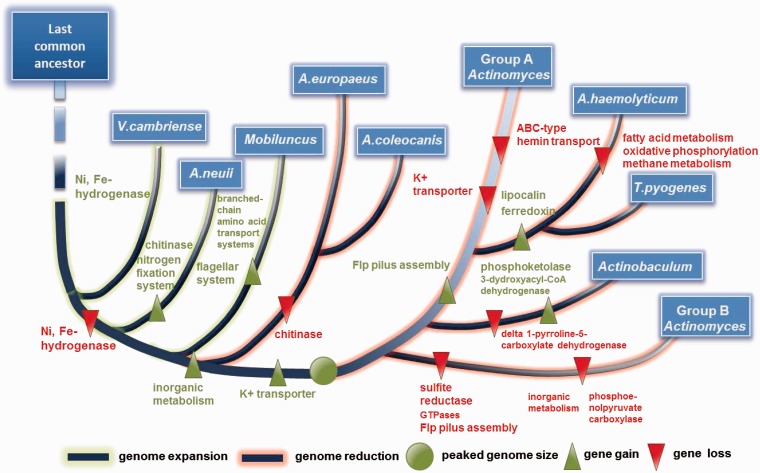
History of *Actinomycetaceae* evolution.

To gain insight into the evolutionary dynamics of the family *Actinomycetaceae*, we then investigated the gene gain/loss events that might have happened during the evolution of the family *Actinomycetaceae* (fig. 5). On the basis of which, the evolutionary history of *Actinomycetaceae* was delineated by comparing the enriched COG terms of extant gene content as well as gained and lost genes of each taxon (fig. 6). The family *Actinomycetaceae* initially experienced a significant genome expansion because a series of gene gain events happened in the early stage of speciation. After comparison of the gene content between taxa, all the species share 998 genes that were probably vertically transferred from the last common ancestor. All groups possess the phosphotransferase systems, which might be necessary for the species to colonize a wide range of hosts and the initiation of abscess (Deutscher et al. 2006; Richards et al. 2014). Interestingly, a large amount of gene loss events happened during lateral evolution which might be due to the adaption in specific environments. For instance, the species of genus *Actinobaculum* have a unique K^+^ transporter and ABC-type hemin transport system (supplementary data sheet S3, Supplementary Material online), which may contribute to the colonization of *Actinobaculum* in urine or blood of hosts.

In conclusion, the current study provided detailed genome landscape of *T. pyogenes* and the critical phylogenetic analyses among the species of the family *Actinomycetaceae* in genomic level. The relationship between *T. pyogenes* and *A. haemolyticum* as well as the classification of *Actinomyces* were further discussed. Moreover, our gene gain/loss analysis revealed a dynamic genome evolution pattern of *Actinomycetaceae*: Lateral gene losses might be the primary mechanism that facilitates the formation of new lineage and specialization in habitat colonization.

## Materials and Methods

### Genome Sequencing and Annotation of *T. pyogenes* TP8

The *T. pyogenes* strain *T. pyogenes* TP8 was isolated from the abscess of body surface of forest musk deer (Miyaluo Farm, Sichuan Province, China), which is an economically important ruminant and categorized as the first class key species protected by the Chinese legislature since 2002 (Guha et al. 2007; Zhao et al. 2011). Genome DNA was isolated with QIAamp DNA Mini Kit (QIAgen) and sequenced on the 454 sequencing platform at the Beijing Genomics Institute and resulted in greater than 15-fold sequencing coverage. High-quality data were assembled by Newbler2.6 and the gaps were then complemented by PCR amplification. Automatic gene prediction and annotation were performed using Glimmer3.02 (Delcher et al. 2007) and BLAST programs (Altschul et al. 1997). The circular genome visualization was performed by CGView (Stothard and Wishart 2005). The obtained entire ORFs were then assigned to the database of COGs (http://www.ncbi.nlm.nih.gov/COG) and KEGG (http://www.genome.jp/kegg/, last accessed June 1, 2014) following the instructions of website, respectively.

### Comparative Genomics Analyses between *A. haemolyticum* and *T. pyogenes*

The genome sequence of *A. haemolyticum* DSM 20595 was reannotated as the process of *T. pyogenes* TP8 mentioned above. The genome wide colinearity between *A. haemolyticum* DSM 20595 and *T. pyogenes* TP8 was determined by BLAST analysis at the nucleotide and amino acid levels. Then the functional similarity of these two species was analyzed by Web Gene Ontology Annotation Plot (WEGO) program (Ye et al. 2006) and by assigning the ORFs to the database of KEGG.

### Genome Clustering

Genome clustering was performed based on the sequences and online program of database of Integrated Microbial Genomes (IMG) (http://img.jgi.doe.gov, last accessed September 29, 2014). The genome sequence of *T. pyogenes* TP8 sequenced here was also submitted to this database under Taxon ID 2558860978. Species of genera *Mobiluncus*, *Actinomyces*, *Varibaculum*, *Arcanobacterium*, *Actinobaculum*, and *Trueperella* were selected to generate the correlation matrix based on whole COG terms (https://img.jgi.doe.gov/cgi-bin/w/main.cgi?section=EgtCluster&page=topPage, last accessed September 29, 2014). And then the data were manually processed by R packages pheatmap software (Kolde 2011) to construct the heatmap in a phylogenetic trait.

### Enrichment of COG Terms and Construction of Phylogenies

The genome sequences of 1 *Varibaculum cambriense*, 16 *Actinomyces*, 4 *Actinobaculum*, 2 *Mobiluncus*, 1 *A. haemolyticum* DSM 20595, and 2 *T. pyogenes* strains were downloaded from the IMG database or NCBI, in addition to that of *T. pyogenes* TP8 sequenced in this study, were used to assess the common gene repertoires based on the COG ID. We consider a COG as a gene, and the common genes were selected within the rule that the same COG ID presents only once in all species. The amino acid sequences were aligned by the algorithm Prank (Löytynoja and Goldman 2005) and transferred it into phylip format by trimAl (Capella-Gutiérrez et al. 2009). Furthermore, the amino acid phylip was transformed into CDS phylip by an in house Perl script. Finally, RaxML v7.3.0 (Stamatakis 2006) was used to construct phylogenetic trees of each common gene using *V. cambriense* as outgroup species. Branch supports of each tree were provided by generating 1,000 bootstrap replicates. All the trees were merged by Maximum Pseudo-likelihood for Estimating Species Trees (MP-EST) in the website http://bioinformatics.publichealth.uga.edu/SpeciesTreeAnalysis/mpest/mpest.php (Liu et al. 2010). The functional divergences between clades were determined by selecting the COG terms that present in at least 80% species of each clade.

### Construction of SNP tree

Combined application of software Mummer (Delcher et al. 2003) and Lastz available at http://www.bx.psu.edu/miller_lab/ (last accessed September 29, 2014) were conducted to identify the overlapped regions exist in these species, and the genome of *A. haemolyticum* was set as the reference. The SNP sites of each species were selected from these regions and tandem assembled to generate one sequence. The program PhyML 3.0 (Guindon et al. 2010) was used to construct the ML tree with a bootstrap value of 1,000 replications in the model of HKY85.

### Gene Gain and Loss

Gene content evolution in the history of *Actinomycetaceae* was reconstructed using COUNT software (Csuros 2010), as previously described (Yutin et al. 2009). The predicted total gene contents, gains, and losses were statistically analyzed in combination with the results of COG enrichments between clades. Each of the phylogenetic clades was tested for the enrichment of specific COG terms relative to other groups.

## Supplementary Material

Supplementary tables S1 and S2, figures S1–S8, and data sheets S1–S3 are available at *Genome Biology and Evolution* online (http://www.gbe.oxfordjournals.org/).

Supplementary Data
