# Supplementary material for: Phylogenomics and Evolutionary Dynamics of the Family Actinomycetaceae
Source: Genome Biol Evol. 2014 Sep 22;6(10):2625–33. doi: 10.1093/gbe/evu211 (PMC4224338; doi:10.1093/gbe/evu211)
Supplement: Supplementary Data [file supp_6_10_2625__index.html]

Phylogenomics and evolutionary dynamics of the family Actinomycetaceae — Phylogenomics and Evolutionary Dynamics of the Family Actinomycetaceae — Supplementary Data 

# Phylogenomics and Evolutionary Dynamics of the Family *Actinomycetaceae*

## Supplementary Data

files

**Files in this Data Supplement:**

- Supplementary Data - pdf file
- Supplementary Data - xlsx file
