# Supplementary material for: Phylogenomics and Evolutionary Dynamics of the Family Actinomycetaceae
Source: Genome Biol Evol. 2014 Sep 22;6(10):2625–33. doi: 10.1093/gbe/evu211 (PMC4224338; doi:10.1093/gbe/evu211)
Supplement: Supplementary Data [file supp_evu211_SI.pdf]

Supplementary figures

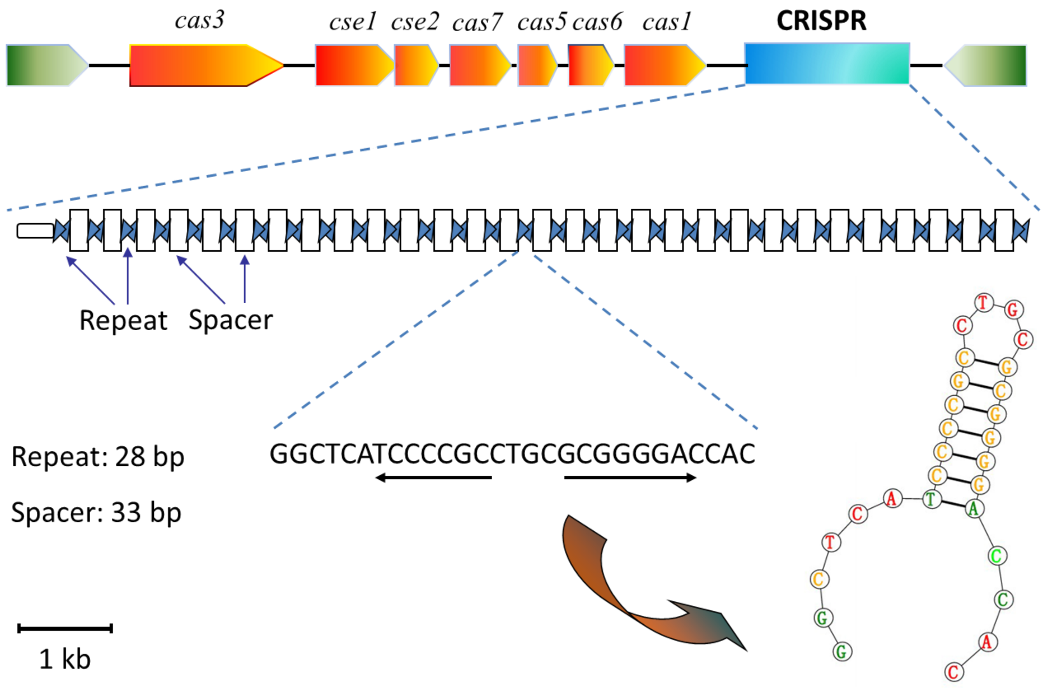

Figure S1. Overview of the CRISPR/Cas system present in *T. pyogenes* TP8. Gene organization is depicted on the top, and the predicted secondary structure of the repeat is shown on the right.

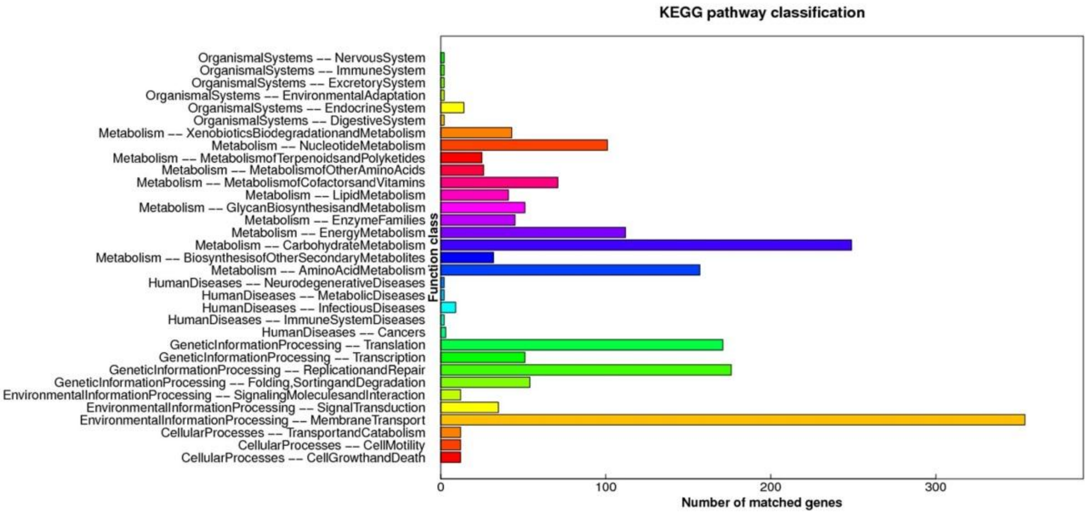

Figure S2. KEGG pathway classification of *T. pyogenes* TP8 based on genome annotation.

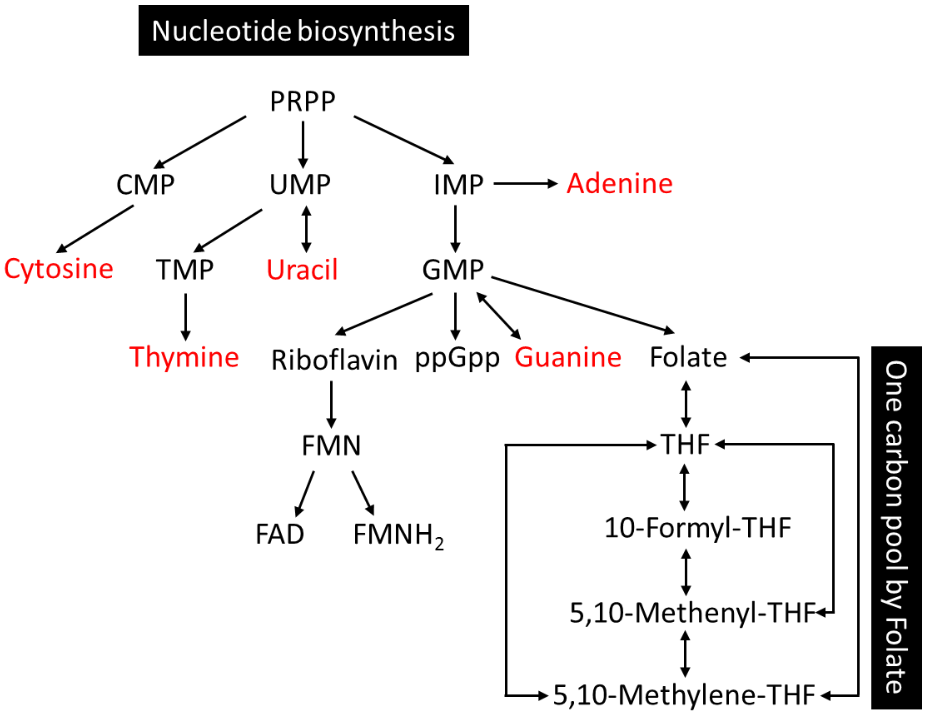

Figure S3. Nucleotide biosynthesis and one carbon pool of *T. pyogenes* TP8.

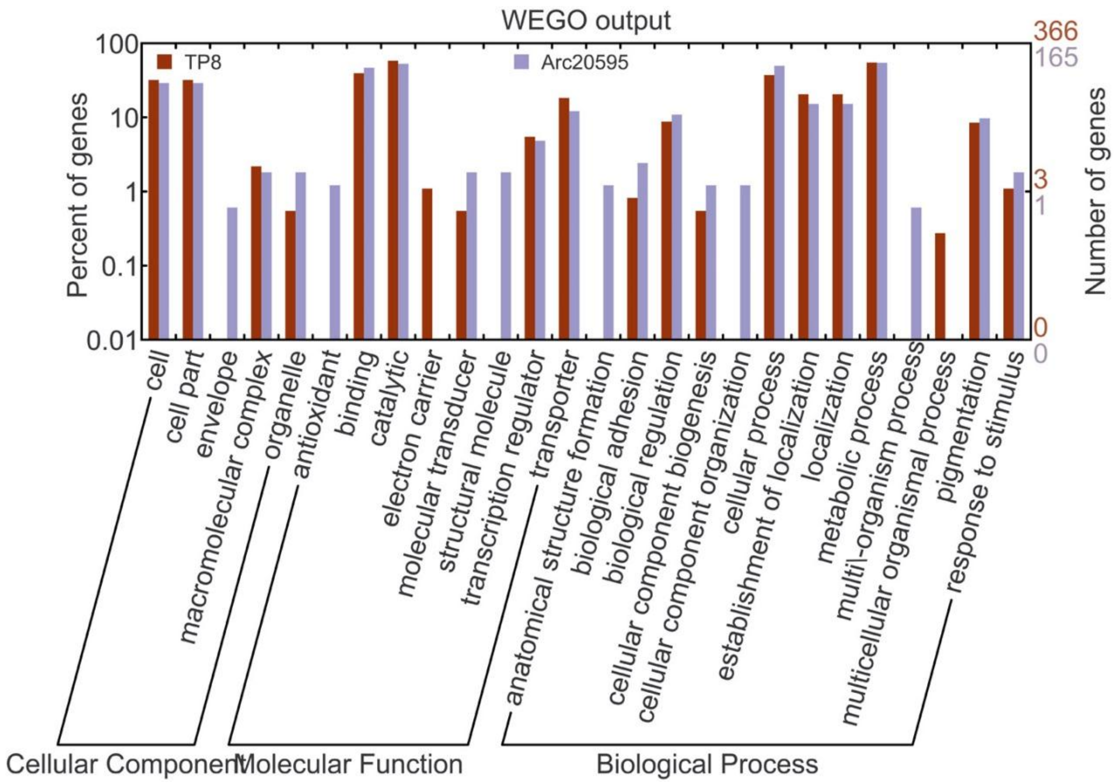

Figure S4. Classification of different Gene Ontology (GO) terms between *T. pyogenes* TP8 and *A. haemolyticum* DSM 20595.

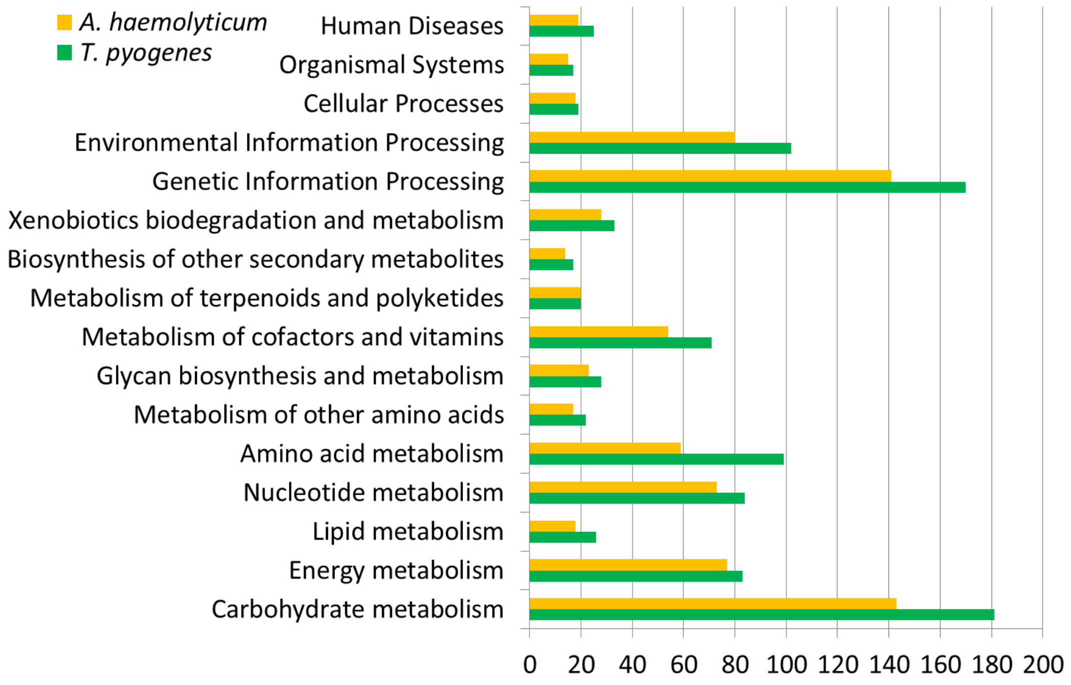

Figure S5. Metabolic differences between *T. pyogenes* TP8 and *A. haemolyticum* DSM 20595. Data were summarized by assigning the ORFs of *T. pyogenes* TP8 and *A. haemolyticum* DSM 20595 in the KEGG pathway database.

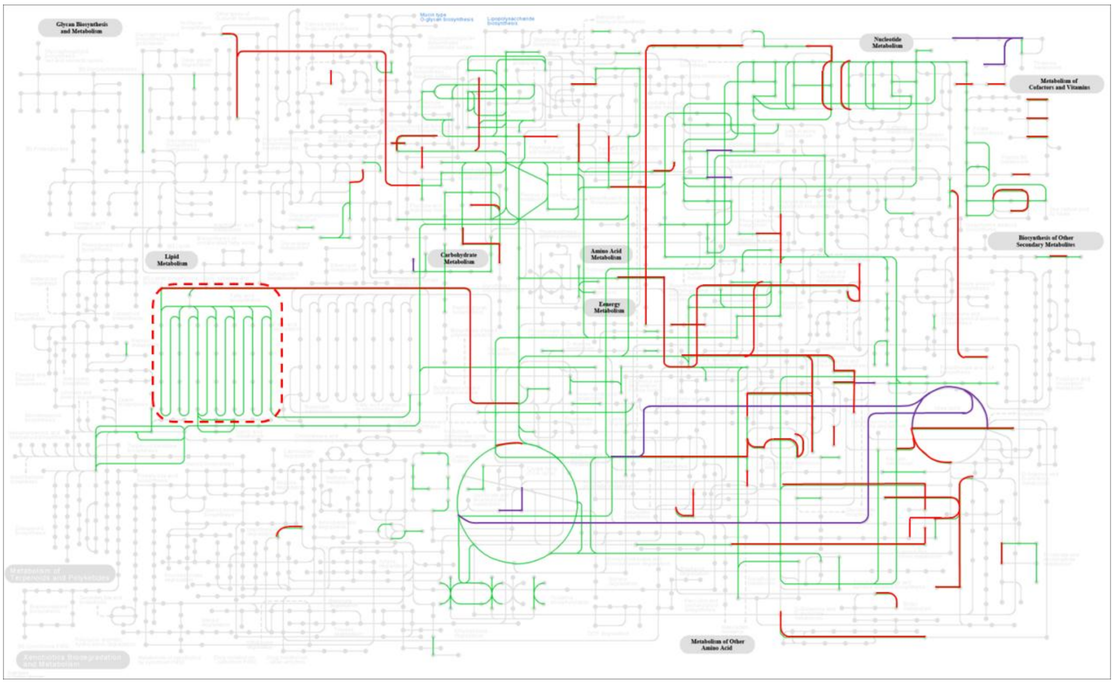

Figure S6. Projection of global metabolic differences between *T. pyogenes* TP8 and *A. haemolyticum* DSM 20595. The global metabolic pathways of *T. pyogenes* TP8 and *A. haemolyticum* DSM 20595 were generated using KEGG Mapper (http://www.genome.jp/kegg-bin/find_pathway_object), respectively. Green line: common metabolic pathway of *T. pyogenes* TP8 and *A. haemolyticum* DSM 20595. Red line: metabolic pathway present in *T. pyogenes* TP8 but absent in *A. haemolyticum* DSM 20595. Violet line: metabolic pathway present in *A. haemolyticum* DSM 20595 but absent in *T. pyogenes* TP8.

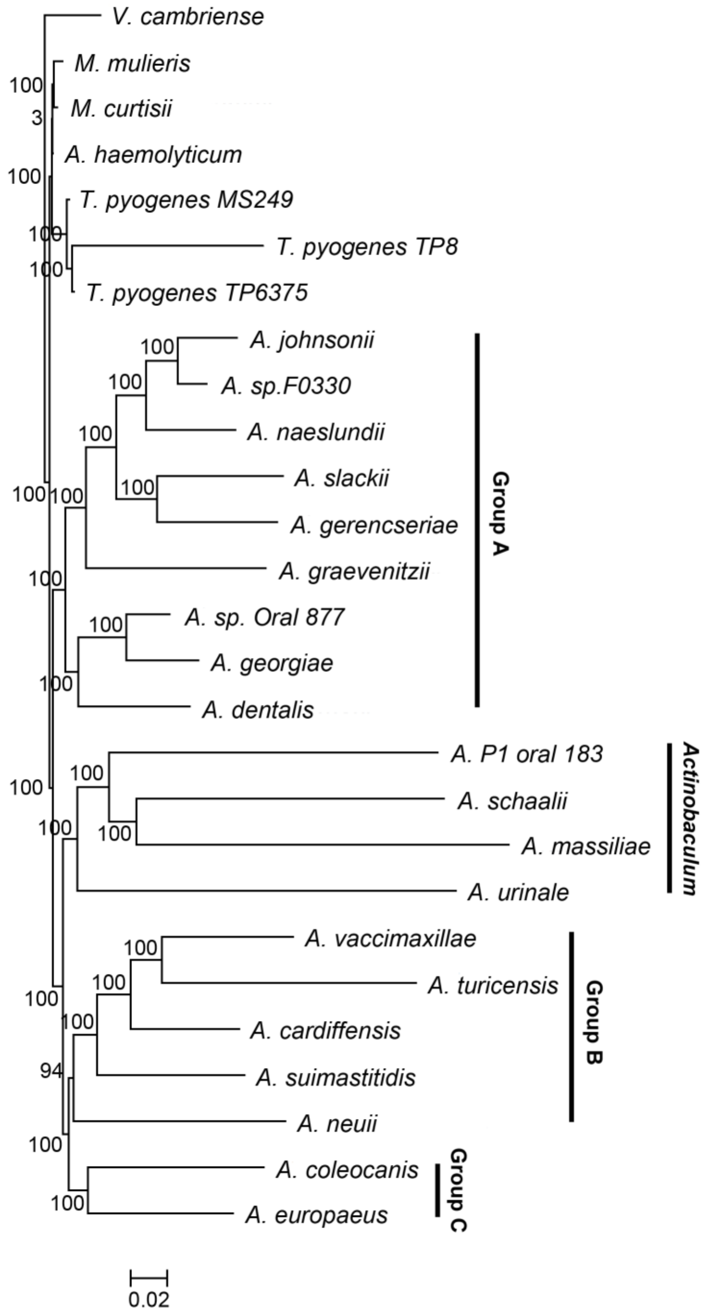

Figure S7. Single nucleotide polymorphisms (SNP) tree of the main species in *Actinomycetaceae*. The phylogenetic tree was constructed based on 267,240 SNPs using Maximum-Likelihood estimation with a bootstrap value of 1000 replications.

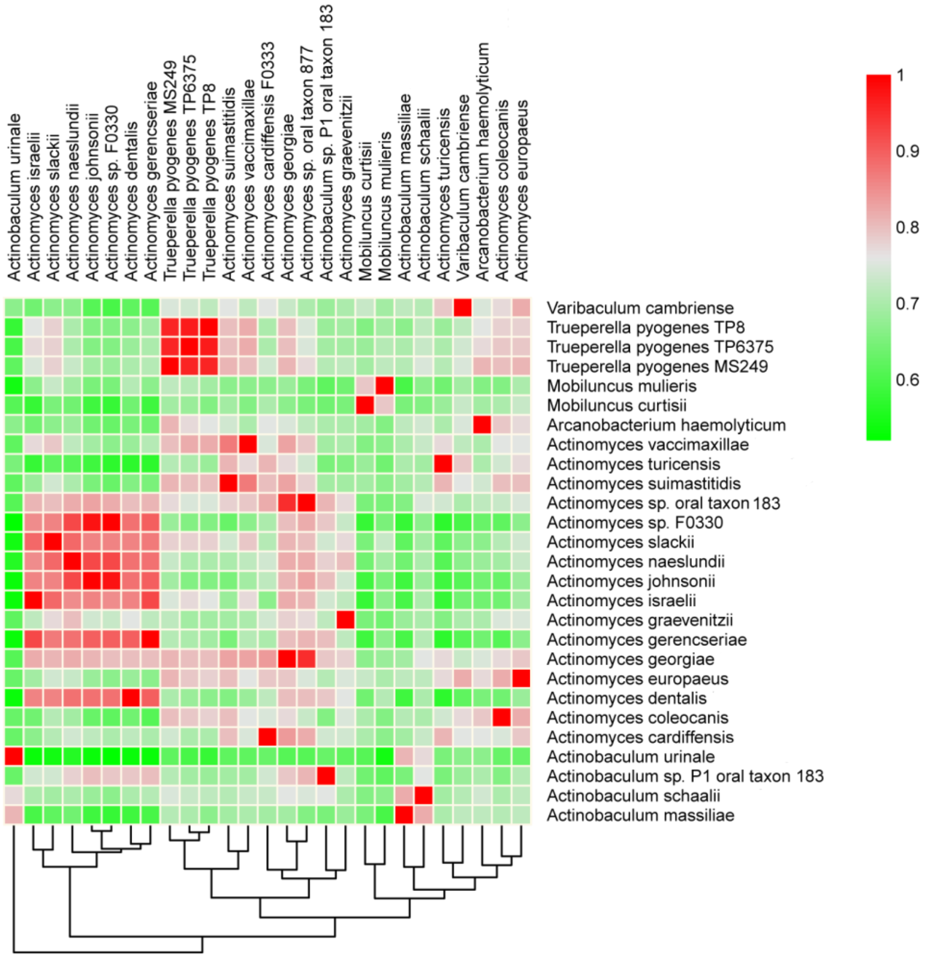

Figure S8. Genome clustering of species in *Actinomycetaceae* based on the whole COGs. The phylogenomic correlation matrix of selected species was automatically generated on the website of IMG (https://img.jgi.doe.gov/cgi-bin/w/main.cgi?section=EgtCluster&page=topPage) and then manually processed by R packages pheatmap (Kolde 2011).

Supplementary tables

Table S1. General features of genome of *T. pyogenes* TP8.

Chromosome organization	Circular
Chromosome size (bp)	2,272,494
GC content (%)	59.58%
GC % of protein coding genes	60.15%
Gene number	2105
Genes total length	2,045,904
Coding content (%)	90.03%
Sense strand gene number	1085
Antisence strand gene number	1020
Average ORF size (bp)	971.93
tRNA number	45
rRNA number	3

Table S2. Genome sequence details.

Species	Source	Tissue/presentation	JGI taxon ID	Genome size (bp)	G+C Content
Actinobaculum					
A. P1 oral 183	human	oral	2541047092	2357424	67.72%
A. massiliae (ACS-171)	human	urogenital tract	2529293003	2021121	60.23%
A. urinale (DSM 15805)	human	urine	2524614758	1920341	50.88%
A. schaalii (DSM 15541)	human	blood	2524614731	2130987	62.25%
					60.27%*
Actinomyces					
A. sp.(F0330)	human	oral	2513237386	3385844	67.10%
A. johnsonii (F0542)	human	oral	2541048002	3321811	67.40%
A. naeslundii (MG1)	human	oral	2502171150	3042856	67.85%
A. slackii (ATCC 49928)	animal	oral cavity	2524614860	3172786	69.99%
A. dentalis (DSM 19115)	human	abscesses of skin	2523533597	3533374	73.07%
A. gerencseriae (DSM 6844)	human	abscesses of skin	2524023137	3420019	70.71%
A. graevenitzii (C83)	human	throat	2513237375	2196917	57.72%
					67.69%*
A. sp. oral 877	human	oral	2541047506	2712944	70.77%
A. georgiae (DSM 6843)	human	oral	2523231019	2500723	69.84%
A. cardiffensis (F0333)	human	oral	2534682138	2188900	61.49%
A. turicensis (ACS-279)	human	vaginal	2534682016	1951528	57.18%
A .vaccimaxillae (DSM 15804)	cow	jaw lesion	2523533527	2338426	57.56%
A. suimastitidis (DSM 15538)	swine	mammary abscess	2523231055	2294128	56.38%
					62.20%*
A. coleocanis (DSM 15436)	animal	urogenital tract	643886017	1719346	49.60%
A. europaeus (ACS-120)	human	urogenital tract	2541046977	1905561	56.65%
					53.13%*
A. neuii (DSM 8576)	human	mammary abscess	2524023166	2270293	56.63%
Arcanobacterium					
A. haemolyticum (DSM 20595)	human	pharyngeal mucosa	646564505	1986154	53.13%
Trueperella					
T. pyogenes (TP8)	animal	abscesses of skin	2558860978	2272494	59.58%
Trueperella pyogenes (MS249)	animal	endometritis	2568526326	2216617	59.85%
Trueperella pyogenes (TP6375)	animal	endometritis	2576861448	2338390	59.50%
Mobiluncus					
Mobiluncus curtisii (ATCC 43063)	human	vagina	648028043	2146480	55.41%
Mobiluncus mulieris (ATCC 35243)	human	vagina	643886108	2398290	55.14%
Varibaculum					
V. cambriense (DSM 15806)	human	postauricular	2524614732	2022936	53.43%

*, mean G+C content value.
